# PrimerMapper: high throughput primer design and graphical assembly for PCR and SNP detection

**DOI:** 10.1038/srep20631

**Published:** 2016-02-08

**Authors:** Damien M. O’Halloran

**Affiliations:** 1 Department of Biological Sciences, The George Washington University, Science and Engineering Hall 6000, 800 22nd St. N.W. Washington DC 20052, USA; 2 Institute for Neuroscience, The George Washington University, 636 Ross Hall, 2300 I St. N.W. Washington DC 20052, USA

## Abstract

Primer design represents a widely employed gambit in diverse molecular applications including PCR, sequencing, and probe hybridization. Variations of PCR, including primer walking, allele-specific PCR, and nested PCR provide specialized validation and detection protocols for molecular analyses that often require screening large numbers of DNA fragments. In these cases, automated sequence retrieval and processing become important features, and furthermore, a graphic that provides the user with a visual guide to the distribution of designed primers across targets is most helpful in quickly ascertaining primer coverage. To this end, I describe here, *PrimerMapper*, which provides a comprehensive graphical user interface that designs robust primers from any number of inputted sequences while providing the user with both, graphical maps of primer distribution for each inputted sequence, and also a global assembled map of all inputted sequences with designed primers. PrimerMapper also enables the visualization of graphical maps within a browser and allows the user to draw new primers directly onto the webpage. Other features of PrimerMapper include allele-specific design features for SNP genotyping, a remote BLAST window to NCBI databases, and remote sequence retrieval from GenBank and dbSNP. PrimerMapper is hosted at GitHub and freely available without restriction.

PCR is a widely employed and indispensable tool for an extensive and ever-growing number of molecular applications^1^. These applications include protocols in diverse fields such as biomedical research, forensic science, as well as phylogenetic analysis^2^,^3^,^4^,^5^. PCR has also been repeatedly tinkered, resulting in creative and versatile modifications. Some of these variants include multiplex PCR, nested PCR, primer walking, DNA cloning, assembly PCR, overlapping PCR, allele-specific PCR, loop-mediated isothermal amplification (LAMP)^6^, and digital PCR^7^. In each case, primer design is the first step in building robust and effective PCR-based experiments. Effective primer design requires numerous calculations, including melting temperature (*T*_*m*_), GC%, self-complementarity, and hairpin formation, which are each derived from a candidate primer’s size and sequence. Manual primer design is laborious and time-consuming and as a result, automated primer design has become a requisite tool in the PCR arsenal^8^,^9^,^10^,^11^,^12^,^13^,^14^,^15^,^16^,^17^,^18^,^19^,^20^.

The diversification of PCR-based methodologies coupled with a rapid expansion in available genomic data has put further demands on the scale and utility of primer design software. In particular, batch primer design has become an important feature of primer design tools to accommodate screening large numbers of genes and datasets. However, when designing primers for large numbers of sequences, automated sequence retrieval and processing becomes important, and furthermore, graphical outputs depicting the map of sequence/primer position is often the best way to rapidly validate primer coverage and distribution. To meets these demands, I describe here a program called PrimerMapper that provides a comprehensive graphical user interface that facilitates the design of robust primers from any number of inputted sequences while providing the user with graphical outputs of primer maps to each inputted sequence, and also a global assembled map of all inputted sequences with designed primers. PrimerMapper also permits the user to visualize each primer sequence map in a browser and allows the user to draw new primers in specific locations directly onto the webpage. Other features of PrimerMapper include primer design tools for SNP genotyping, a remote BLAST facility, and also remote sequence retrieval from dbSNP and GenBank.

PrimerMapper will be helpful for researchers working with large datasets where primers must be efficiently designed for many genes or SNPs, as well as for various PCR-based applications including primer walking, nested PCR, sequence-specific probe construction, and assembly PCR reactions, where graphical outputs can quickly help users determine primer distribution.

## Results and Discussion

**Figure 1:**
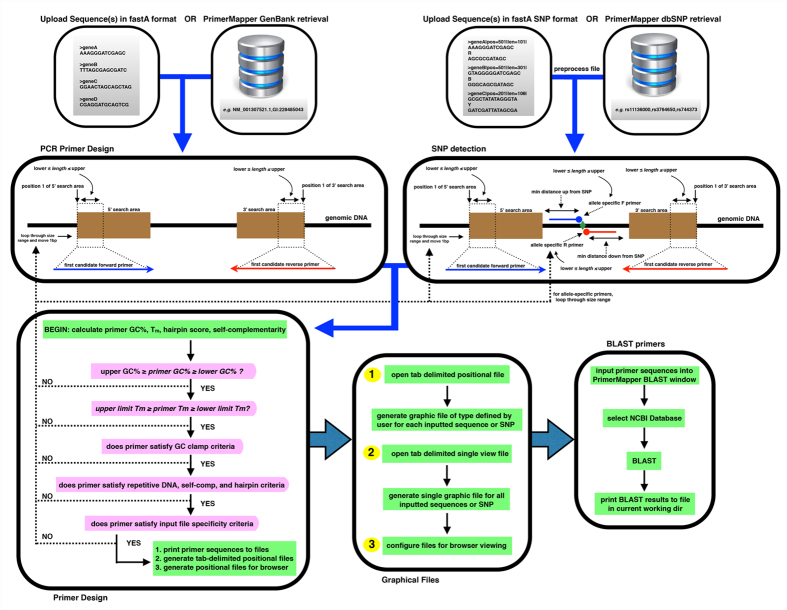
Flowchart of the PrimerMapper process. Data can be uploaded into PrimerMapper from a local file or from NCBI’s GenBank^22^ or dbSNP^23^ databases. SNP sequence data is preprocessed by PrimerMapper prior to execution to collect the SNP position and type from the header field, and also to remove whitespace from the header field so as to preserve the entire sequence name. The user sets the various parameters for primer design for DNA sequence or SNP sequences such as primer maximum and minimum lengths, GC%, melting temperature (*T*_*m*_), etc. PrimerMapper will then calculate hairpin and self-complementarity scores and only return primers whose scores meet specific thresholds. Repetitive sequence consisting of 5 or more mononucleotide repeats or more than 4 dinucleotide repeats is excluded from primer design by default (this can be changed by the user). Once all criteria are met, PrimerMapper will print the primer sequence and features to a file, and also generate files that mark the position and length of each primer within each sequence. These positional files are used to generate the graphical outputs for each sequence. For multiplex PCR reactions, it is useful to examine potential cross-complementarity between all primer pairs-to achieve this PrimerMapper can also implement a combinations without replacements algorithm (*n* choose *k*) for all primers to calculate cross-complementarity primer-dimer scores. Primer sequence(s) can then be inputted into the BLAST window to generate BLAST reports for each primer at a user-defined NCBI database.

Generating graphics that map each designed primer onto the target sequence provides easy and fast validation controls for researchers to examine the distribution and position of each primer. PrimerMapper (http://dohalloran.github.io/PrimerMapper/) can automate sequence retrieval and primer design for any number of sequences while returning maps of primers along each sequence that can be visualized as image files or within a browser (see Fig. 1 for overview).

### User Interface

**Figure 2:**
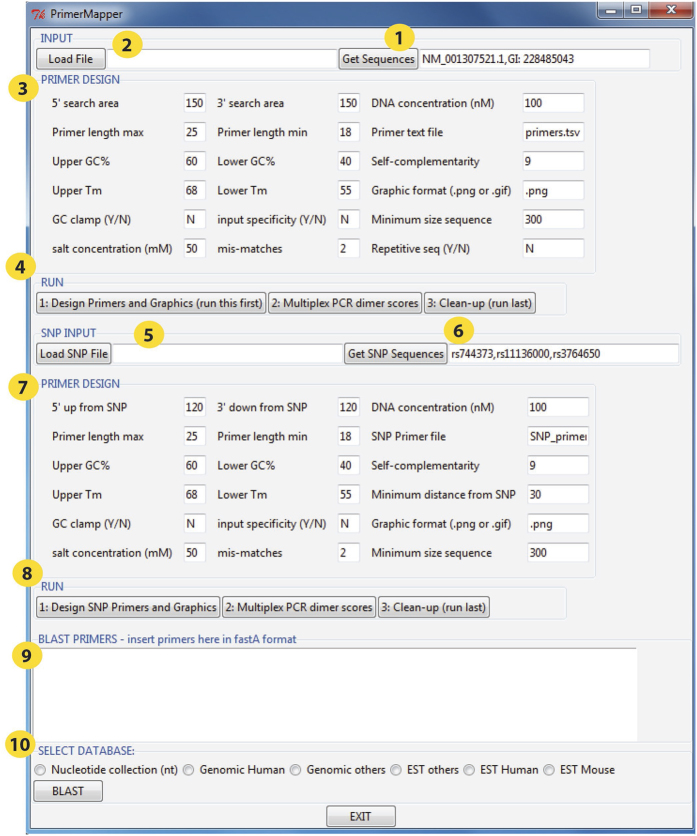
PrimerMapper user interface. The PrimerMapper user interface is divided into *three* sections that can be executed independently. The *first* section is for general primer design from DNA sequences in fastA format that can be uploaded by clicking the “Get Sequences” button which will collect fastA formatted sequences from NCBI’s GenBank (yellow circle, number 1), or locally by clicking the “Load File” button (yellow circle, number 2). The Primer design criteria are populated in the textboxes within the “PRIMER DESIGN” frame (yellow circle, number 3). Primer design then begins by clicking the buttons within the “RUN” frame (yellow circle, number 4). The user must start with “1: Design Primers”, followed by “2: Multiplex PCR dimer scores” or “3: Clean-up”. The *second* section of the interface is for “SNP INPUT”. Similar to the *first* section, the data can be uploaded locally (yellow circle, number 5) or remotely from dbSNP by entering appropriate accession number(s) (yellow circle, number 6). Next the primer design criteria are populated within the “PRIMER DESIGN” frame (yellow circle, number 7), followed by program execution by clicking the buttons 1 to 3 in the “RUN” frame (yellow circle, number 8). The *third* section is the BLAST window at the bottom of the interface (yellow circle, number 9); here the user can input any number of fastA formatted primer sequences and BLAST against a specific database at NCBI (yellow circle, number 10). The results from the BLAST report are printed to a text file in the current working directory.

The user interface (Fig. 2) is divided into *three* components that can be executed independently. The *first* section is for general primer design from DNA sequences in fastA format^21^ that can be uploaded remotely from NCBI’s GenBank^22^ by clicking the “Get Sequence” button (Fig. 2, circle 1) or locally by clicking the “Load File” button (Fig. 2, circle 2). For remote retrieval, PrimerMapper uses the e-utilities feature from NCBI (Supplementary Table S1). Sample sequence accession numbers are populated in the “Get Sequence” textbox – these accession numbers can be deleted by the user and updated. The Primer design criteria are populated in the textboxes within the “PRIMER DESIGN” frame (Fig. 2, circle 3). PrimerMapper will collect the primer design criteria defined by the user such as primer maximum and minimum lengths, GC%, and melting temperature (*T*_*m*_), as well as the five-prime (5′) and three-prime (3′) search windows. The search windows are the areas across which PrimerMapper will scan for appropriate primers that meet the user’s requirements; for example, a five-prime (5′) search area of ‘150’, will result in PrimerMapper searching the first 150bp of each sequence for an appropriate primer sequence. PrimerMapper will then calculate hairpin and self-complementarity scores for each primer, and only return primers whose scores are above a minimum threshold. Thresholds were determined by PCR validation experiments and also *in silico* testing. By default, repetitive sequences are excluded from primer design – this is defined as more than 5 mononucleotide repeats or more than 4 dinucleotide repeats. However, if the user wishes to design primers from repetitive sequences, ‘Y’ can be entered in the ‘repetitive sequence’ option box (see Fig. 2). A three-prime (3′) GC clamp can also be specified by the user for each primer (this is not the case for allele specific primers). PrimerMapper also includes a primer specificity detection feature with mismatch options. To use this feature the user can enter “Y” in the “input specificity” textbox, and enter the number of allowed mismatches, if any, in the “mis-matches” textbox – this feature will ensure that each primer is specific (apart from permitted mismatches) to the entire input file uploaded by the user. After all the primer design parameters are completed by the user, PrimerMapper is then executed by clicking the button within the “RUN” frame (Fig. 2, circle 4). Once all criteria are met, PrimerMapper will print the primer sequence and features to a file, and also generate text based files that mark the position and length of each primer within each sequence. These positional text files are used to generate the graphical maps for each sequence. If the user clicks the “Multiplex PCR dimer scores” button, PrimerMapper also implements a combinations without replacements algorithm (*n* choose *k*: equation 1) for all primers (both forward and reverse) to calculate cross-complementarity primer-dimer scores. The user must start with “1: Design Primers”, followed by “2: Multiplex PCR dimer scores” or “3: Clean-up”. The “Clean-up” button can be clicked at the end by the user to remove temporary files from the current working directory used in the generation of graphical outputs.

The *second* section of the interface is the SNP INPUT component. Similar to section 1 above, the data can be uploaded locally in rs_fastA format (Fig. 2, circle 5) or retrieved remotely (Fig. 2, circle 6) from NCBI’s dbSNP^23^. SNP sequence data is preprocessed by PrimerMapper to collect the SNP position and type from the header field. Next, the primer design criteria are populated within the “PRIMER DESIGN” frame (Fig. 2, circle 7), followed by program execution after clicking buttons 1 to 3 within the “RUN” frame (Fig. 2, circle 8). PrimerMapper’s SNP primer design includes the design of allele-specific primers. Allele-specific PCR is a PCR-based method used to detect known SNPs^24^,^25^. In this protocol, the specific primers are designed to permit amplification by DNA polymerase only if the nucleotide at the 3′-end of the primer perfectly complements the base at the wildtype or polymorphic site.

The *third* section is the BLAST^26^ window to NCBI databases at the bottom of the interface (Fig. 2, circle 9); here the user can input any number of fastA formatted primers and BLAST against a specific database at NCBI (Fig. 2, circle 10). The database is selected by the user and includes the following: nucleotide collection, genomic human, genomic others, EST others, EST human, EST mouse. The results of the BLAST analysis are printed to a text file in the current working directory. The BLAST feature is driven by a separate script called ‘web_blast.pl’ that should be in the same PATH or directory as the driver script mentioned above.

### Output files

**Figure 3:**
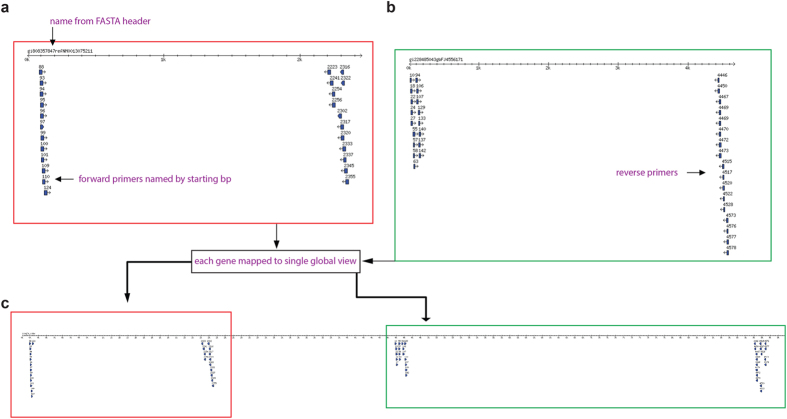
PrimerMapper graphical outputs. (**a,b**) A sample graphical output generated by PrimerMapper for DNA based sequences in fastA format is shown. The primers designed to each sequence (in this case, two sequences), is converted to a graphical output with each primer represented by a blue glyph arrowed in the direction of synthesis. Each primer is named by its starting position and the fastA header from each sequence is at the top of the scaled graphic. The number line represents a scaled version of the input sequence in base pairs. **(c)** PrimerMapper also generates a concatenated graphic that assembles each sequence (adopting the order followed in the inputted file) and its designed primers into a single view so as to quickly and easily validate the distribution of primers across all sequences.

**Figure 4:**
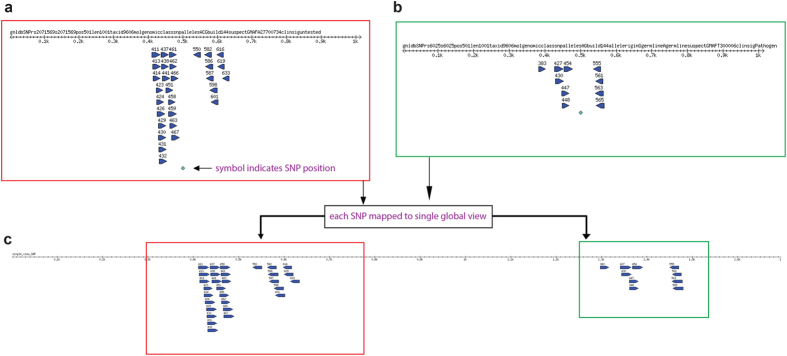
PrimerMapper graphical outputs for SNP based sequences. (**a,b**) A sample SNP sequence graphical output generated by PrimerMapper is shown. Primers spanning the SNP (which is denoted by a green symbol) are mapped onto their derived sequence. Each primer is named by its starting position and the fastA header from each sequence is at the top of the scaled graphic. The number line represents a scaled version of the input sequence in base pairs. **(c)** A single assembled view of all inputted SNP sequences and their primers is also generated from SNP data by PrimerMapper.

An example of a primer sequence map generated by PrimerMapper for DNA based sequences in fastA format is shown in Fig. 3. The primers designed to each inputted sequence (in this case only two sequences), is converted to a graphical output with each primer represented by a blue glyph arrowed in the direction of synthesis (Fig. 3a,b). Each primer is named by its starting position and the fastA header from each sequence is at the top of the scaled graphic. The number line represents a scaled version of the input sequence in base pairs. PrimerMapper can also generate a concatenated map that assembles each sequence (adopting the order followed in the inputted file) and its designed primers into a single map so as to quickly and easily validate the distribution of primers across all sequences (Fig. 3c). This approach is also implemented for SNP based sequences (Fig. 4a–c) where primers spanning the SNP (which is denoted by a green symbol – see Fig. 4a) are mapped onto their derived sequence. A single assembled view of all inputted SNP sequences and their primers can also be generated from SNP data by PrimerMapper (Fig. 4c).

**Figure 5:**
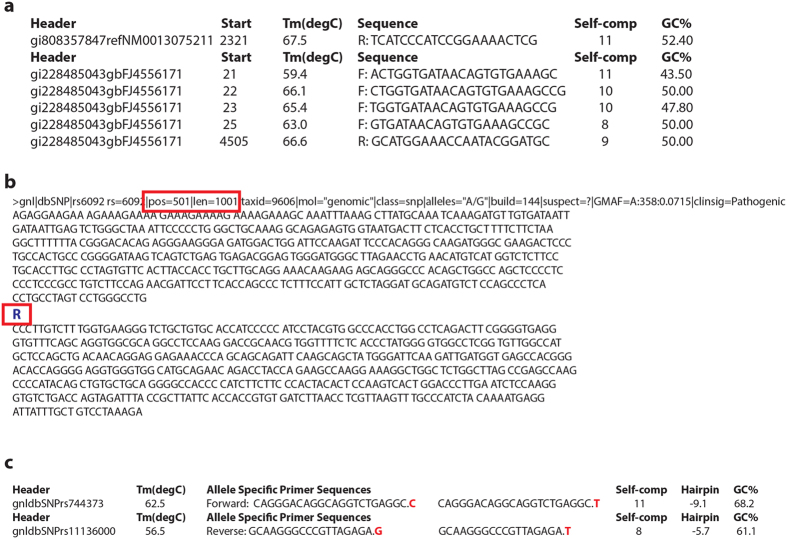
PrimerMapper text based outputs and input SNP sequence requirements. (**a**) PrimerMapper generates tab separated value (TSV) based text files that lists the designed primers and their corresponding features from each sequence. (**b**) In the case of SNP data, PrimerMapper pre-processes all input by collecting the SNP location and SNP type from the rs_fastA formatted input. These features are highlighted by red rectangles. If the SNP sequence data is not remotely retrieved from dbSNP^23^ , the user can locally upload data, however, the header for each sequence must be unique and contain the SNP location and sequence length as formatted within the red rectangle i.e. “pos = 501|len1001”. Furthermore, the standard SNP sequence format must be adopted with the SNP type e.g. “R” or “Y” etc. inserted within each sequence. (**c**) PrimerMapper returns a TSV file from SNP data depicting the allele-specific primers for each SNP and the appropriate wildtype and polymorphic primers specific to each SNP, as well as their basic features i.e. sequence header, *T*_*m*_, Self-complementarity score, ΔG hairpin score, and GC%.

PrimerMapper also generates tab separated value (TSV) based files that list the designed primers and their corresponding features from each inputted sequence (Fig. 5a). In the case of SNP data, PrimerMapper preprocesses all input by collecting the SNP location and SNP type from the rs_fastA formatted input. These features are highlighted in Fig. 5b by red rectangles. If the SNP sequence data is not remotely retrieved from dbSNP, the user can locally upload their own data; however, the header for each sequence must be unique and contain the SNP location and sequence length as indicated within the red box i.e. “pos = 501|len1001”. Furthermore, the standard SNP sequence format must be adopted for the SNP type, e.g. “R” or “Y”, inserted within each sequence where standard IUPAC notation^27^ is applied: R = A/G, Y = C/T, M = A/C, K = G/T, W = A/T, S = C/G, B = C/G/T, D = A/G/T, H = A/C/T, V = A/C/G, N = A/C/G/T. As well as generating a TSV file similar to Fig. 5a, PrimerMapper will also generate a TSV file from SNP data similar to Fig. 5c depicting the allele specific primers for each SNP and the appropriate wildtype and polymorphic primer specific to each SNP, as well as the basic features for each primer *e.g.* sequence header, *T*_*m*_, Self-complementarity score (score should be below 10), hairpin score (ΔG score closer to zero is better), and GC%.

### Browser visualization of primer map

**Figure 6:**
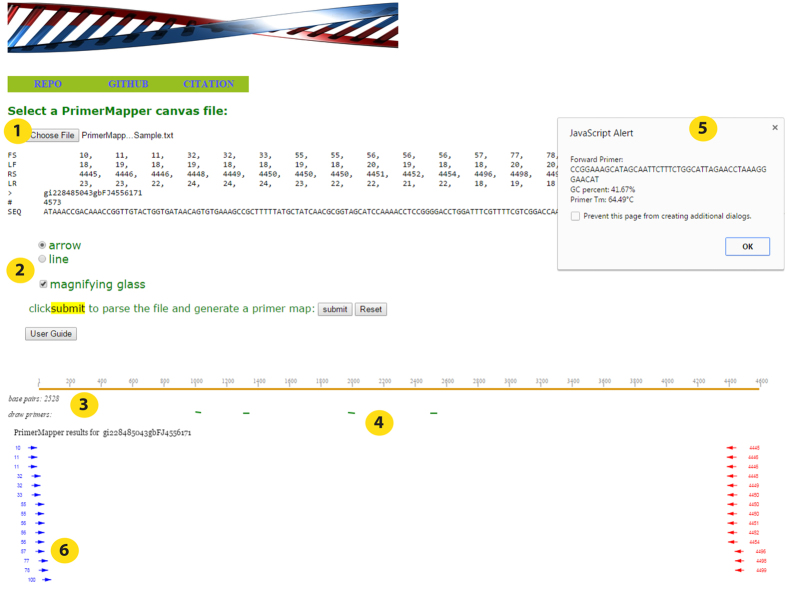
Visualization of primer map in a browser. A sample graphical output generated by PrimerMapper for browser visualization is shown. A canvas text file for each sequence is generated by PrimerMapper and these files can then be opened directly in a browser by clicking the “primermapper.HTML” file and loading the data. A sample configured text file is included in the download called “PrimerMapper_Sample.txt” which can be directly loaded into “primermapper.HTML” (circle 1). Primers can be visualized as arrows or lines and also a ‘magnifying glass’ can be checked so as to magnify the primer names by moving the mouse over the primers within the primer-map canvas (circle 2). The user can also get the base pair position along the sequence (circle 3) by moving the mouse under the sequence map. The user can also draw new primers on the canvas (circle 4) which will return a JavaScript popup box with the DNA sequence, GC%, and primer *T*_*m*_ values of the sequence corresponding to the line drawn by the user (circles 4 and 5). If the line is drawn from left to right, a forward primer is returned, and if the line is drawn from right to left, a reverse complemented primer is returned in the popup box. The user can also click the ‘User Guide’ button to get an overview of each feature.

PrimerMapper will generate positional text files that are configured for browser visualization using the script ‘cols_to_rows.pl’. This script must be placed in the current working directory or specified PATH. A folder called “Browser_PrimerMapper” will be generated in the current working directory by PrimerMapper and a file for each primer/sequence map is placed inside this folder. These files can be opened from within “primermapper.html” under ‘choose file’ (Fig. 6, circle 1). Primers can be visualized as arrows or lines, and also a “magnifying glass” checkbox can be ticked so as to magnify the primer names by moving the mouse over the primers within the primer-map canvas (Fig. 6, circle 2). The user can then click the “submit” button and the primer/sequence map is generated, which allows the user to get the base pair position along the sequence by moving the mouse under the sequence map (Fig. 6, circle 3). The user can also draw new primers directly onto the canvas (Fig. 6, circle 4) which will return a JavaScript popup box with the DNA sequence, GC%, and primer *T*_*m*_ values of the sequence that corresponds to the line drawn by the user (Fig. 6, circle 5). If the line is drawn from left to right, a forward primer is returned, and if the line is drawn from right to left, a reverse complemented primer is returned in the popup box. The primers are graphically represented as arrows in Fig. 6 (circle 6). This feature not only provides a graphical output to visualize the primer/sequence map but also allows the user to draw new primers in specific positions, for example, perhaps flanking a SNP, or perhaps relative to others primers within the primer map for nested PCR experiments.

### Testing

**Figure 7:**
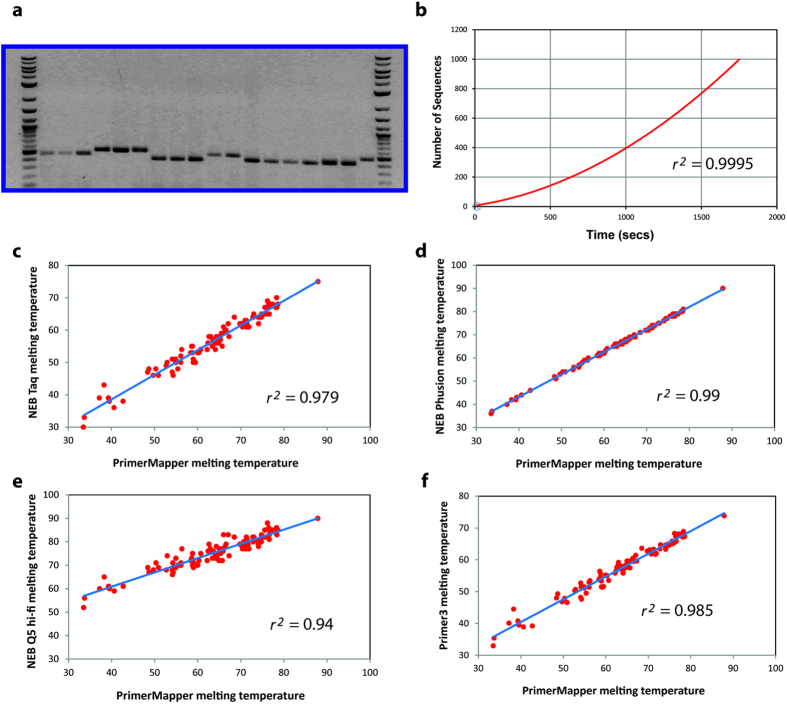
Validation and testing of PrimerMapper. (**a**) Primers were designed using PrimerMapper to span the *C. elegans* transcript, ZK5204a, and PCR products from a series of PCR reactions using these primers were examined by gel electrophoresis. Primer sequences are displayed in Table 1. Robust bands of the correct size were obtained from each PCR. The first and last lanes are a GenRuler DNA ladder mix (Thermo Fisher Scientific, Waltham, MA). Primer parings starting from the left second lane were: F2 + R1, F2 + R2, F2 + R3, F2 + R4, F2 + R5, F2 + R6, F3 + R1, F3 + R2, F3 + R3, F3 + R4, F1 + R2, F1 + R3, F1 + R4, F1 + R5, F1 + R6, F1 + R8, F3 + R9, F5 + R6. (**b**) Files containing different numbers of sequences (2, 10, 20, 50, 100, 150, 200, 1,000) were provided as input to PrimerMapper and ran using default settings to generate primer text files for each sequence and graphic file generation input text files (i.e. Step 1 of execution – see Fig. 2). The resulting data is plotted with time (seconds) on the x-axis and the number of sequences on the y-axis. Fitting the relationship between sequence number and run-time with a quadratic equation yields an R^2^ value of 0.9995. (**c–f)** Comparison of the melting temperatures obtained for PrimerMapper from 100 randomly generated primers (18–30bps in size) with that of the NEB calculator for NEB *Taq* DNA Polymerase **(c)**, NEB Phusion^®^ Polymerase (**d**), NEB Q5^®^ Hi-Fi Polymerase **(e)**, and with Primer3^16^ (**f**).

Validation tests were performed for PrimerMapper via *in silico* analysis as well as PCR based experimentation. PCR based tests are shown in Fig. 7a, and the corresponding primer sequences are listed in Table 1. PCR reactions using primers designed by PrimerMapper that span the *C. elegans* transcript, ZK5204a, were performed, and the resulting products are presented in Fig. 7a. In each case bands of the correct size were obtained from each PCR. Run-time testing was also performed for PrimerMapper (Fig. 7b); run-time tests using files containing various numbers of sequences (2, 10, 20, 50, 100, 150, 200, and 1,000) were provided as input to PrimerMapper, and ran using default settings to generate primer files and text based positional files by executing the first step in the “RUN” frame of DNA based local fastA formatted sequences (see Fig. 2, yellow circle number 4). The relationship between run-time and sequence number was best fit with a quadratic equation. To compare the primer melting temperatures calculated by PrimerMapper with other algorithms, we generated 100 random primers that varied in size from 18-30bps and plotted their melting temperature against the melting temperatures obtained using other algorithms (Fig. 7c–f). Firstly, the melting temperature obtained by PrimerMapper was compared with the NEB calculator for NEB *Taq* DNA polymerase (Fig. 7c; *r*^2^ = 0.979). The NEB calculator uses the algorithm defined by SantaLucia^28^ and is salt corrected as described by Owczarzy *et al.*^29^ for *Taq* DNA polymerase buffer. Next, we compared PrimerMapper with the NEB calculator for NEB Phusion^®^ polymerase (Fig. 7d; *r*^2^ = 0.99); the algorithm for Phusion^®^ polymerase is defined by Breslauer *et al.*^30^ and salt corrected to the appropriate Phusion^®^ polymerase buffer conditions as described by Schildkraut^31^. The melting temperature correlations are also plotted for PrimerMapper versus NEB Q5^®^ Hi-Fi polymerase (Fig. 7e; *r*^2^ = 0.94), which uses the algorithm by SantaLucia^28^ and is salt corrected as described by Owczarzy *et al.*^29^ for the Q5^®^ buffer system. Finally, the primer melting temperatures for PrimerMapper were compared to that of Primer3^16^ (Fig. 7f; *r*^2^ = 0.985) which by default uses the algorithm by Breslauer *et al.*^30^. In each case, there were robust correlations observed between the melting temperatures calculated by PrimerMapper and each algorithm, with the highest correlation observed for PrimerMapper to that of the NEB calculator for NEB Phusion^®^ polymerase (Fig. 7d; *r*^2^ = 0.99).

## Conclusions

In order to efficiently design large numbers of primers from different data types, automated sequence retrieval and processing becomes critical. Of equal importance is an ability to quickly scan and validate the coverage and position of designed primers. Many primer design tools such as Primer3^16^, BatchPrimer3^9^, Primer-Blast^20^, PrimerDesign-M^32^,^33^, PrimerView^34^ and PerlPrimer^14^ provide effective ways to analyze large datasets. PrimerMapper builds upon these developments to provide a central resource that combines the key features of these tools while also offering a new layer of design and visualization that are not offered by any other primer design tools. To automate the process of bulk primer design, PrimerMapper offers sequence retrieval and processing options from GenBank and dbSNP. In order to quickly validate the density and coverage of designed primers, PrimerMapper returns primer maps for each sequence as an image file, and also generates a single concatenated primer map of all sequences with their derived primers; this latter feature is not offered by other software and provides a fast and effective way to examine primer distribution across contigs or linked genes for primer walking or sequencing experiments. Another unique innovation of PrimerMapper is the ability to view these primer maps within a browser where new primers can quickly and easily be drawn by the user directly onto the webpage. This feature allows the user to design primers that may flank a known SNP or alternatively generate new primers in unique positions or relative to other primers for nested PCR experiments. PrimerMapper’s interface offers numerous other features including remote BLAST options similar to the software, Primer-Blast^20^, as well as generating primer dimer scores for each primer pairing. Taken together, PrimerMapper attempts to bring together key features from numerous primer design tools into a single program while adding new layers of design that enable primer design *en masse* from any number of DNA or SNP sequences.

## Methods

### Implementation

The graphical user interface of PrimerMapper is written using Perl Tkx, which comes standard with ActiveState’s Perl distribution, as well as JavaScript (jQuery and Fabric.js), HTML, and CSS. PrimerMapper also uses the Bioperl^35^ dependencies: Bio:SeqIO, Bio::Graphics, and Bio::SeqFeature::Generic, to parse sequences and generate graphical outputs. All dependencies are freely available from CPAN. A full list of webpages for all software used is documented in Supplementary Table S1. PrimerMapper is a package with a constructor subroutine called “new” that enables the user to execute the module by instantiating a PrimerMapper object. Subroutines from PrimerMapper are exported from the package into a driver script called ‘PrimerMapper_driver.pl’. Browser visualization of the primer map has been validated using Google Chrome and requires the script called ‘cols_to_rows.pl’ to be in the same PATH or directory as the driver script mentioned above (PrimerMapper_driver.pl). To examine potential cross-complementarity between all primer pairs, PrimerMapper implements a combinations without replacements algorithm (*n* choose *k*) for all primers (both forward and reverse) to calculate cross-complementarity primer-dimer scores and print these values to a TSV file using the following formula:


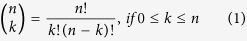


where, *n* is the set of all primers designed by PrimerMapper and *k* is the number of primers chosen (i.e. 2) for each *k*-combination. This implementation slows down substantially as the number of primers designed by PrimerMapper increases (or as the number of sequences increases). Features of the basic algorithm for PrimerMapper are shown in detail in Fig. 1, some of which have been described previously^17^,^19^,^34^ including calculations for primer *T*_*m*_^8^,^19^ which were selected based upon validation experiments performed using primers designed using these calculations (Fig. 7c–f).

### DNA isolation and PCR

*Caenorhabditis elegans* wildtype (N2) strain was maintained at 20 °C using standard procedures on NGM plates seeded with *E. coli* strain OP50^36^. *C. elegans* DNA was isolated by harvesting a mixed population of animals collected in a 1.5 ml tube. 200 μl of lysis buffer (60 g/ml proteinase K, 10 mM Tris-Cl, pH 8.3, 50 mM KCl, 2.5 mM MgCl_2_, 0.45% IGEPAL, 0.45% Tween−20, 0.01% gelatin) was added to the tube and then placed in a freezer at −80 °C for 10 mins followed by incubation at 60 °C for 1 hr followed by 95 °C for 15 mins. Tubes were then centrifuged at 13,000 rpm for 1 min in a bench top centrifuge and 50 μl gDNA supernatant isolated for PCR. Primer pairs used for *C. elegans* PCR reactions are displayed in Table 1 and PCR reactions performed with Taq DNA polymerase from NEB (M0273L) using the following cycling conditions: 95 °C for 2 mins, 95 °C for 30 sec, 55 °C for 30 sec, and 72 °C extension for 1min for 35 cycles. The final PCR products were electrophoresed on 1.5% agarose gels.

## Additional Information

**How to cite this article**: O’Halloran, D. M. PrimerMapper: high throughput primer design and graphical assembly for PCR and SNP detection. *Sci. Rep.* **6**, 20631; doi: 10.1038/srep20631 (2016).

## Supplementary Material

Supplementary Video

Supplementary Information

## Figures and Tables

**Table 1:** DNA sequences of primers used in PCR validation experiments.

Primer Name	DNA Sequence 5′ → 3′
F1_83	TCCCCGAAAATCTTCAGTG
F2_84a	CCCCGAAAATCTTCAGTGT
F3_84b	CCCCGAAAATCTTCAGTG
F4_129a	TTCACCGTCCACAGGCAAA
F5_129b	TTCACCGTCCACAGGCAA
R1_554	TAACCTGTGGACGAGGTGGA
R2_555a	AACCTGTGGACGAGGTGGA
R3_555b	TAACCTGTGGACGAGGTGG
R4_555c	ATAACCTGTGGACGAGGTG
R5_556	TAACCTGTGGACGAGGTG
R6_557a	CATAACCTGTGGACGAGGT
R7_557b	CATAACCTGTGGACGAGG
R8_614	ATGGTCTCAACTGCACCCAT
R9_615	TGGTCTCAACTGCACCCA
