# Supplementary material for: PrimerMapper: high throughput primer design and graphical assembly for PCR and SNP detection
Source: Sci Rep. 2016 Feb 8;6:20631. doi: 10.1038/srep20631 (PMC4745053; doi:10.1038/srep20631)
Supplement: Supplementary Information [file srep20631-s2.pdf]

SUPPLEMENTARY DATA

Manuscript Title: PrimerMapper: high throughput primer design and graphical assembly for PCR and SNP detection

Author: Damien M. O'Halloran

Supplementary Table S1. List of software and websites referenced

Supplementary Video S1. User-guide to installing and running PrimerMapper.

Supplementary Table S1. List of software and websites referenced

Program or site	Webpage
dbSNP	http://www.ncbi.nlm.nih.gov/projects/SNP/
ActivePerl	http://www.activestate.com/activeperl
BioSeqIO	http://search.cpan.org/dist/BioPerl/Bio/SeqIO.pm
BioGraphics	http://search.cpan.org/~lds/Bio-Graphics-2.39/lib/Bio/Graphics.pm
BioSeqFeature	http://search.cpan.org/dist/BioPerl/Bio/SeqFeature/Generic.pm
CPAN	http://www.cpan.org/
GenBank	http://www.ncbi.nlm.nih.gov/genbank/
e-utilities	http://www.ncbi.nlm.nih.gov/books/NBK25500/
jQuery	https://jquery.com/
Fabric.js	http://fabricjs.com/
NEB Tm calculator	http://tmcalculator.neb.com/#!/

Supplementary Video S1. User-guide to installing and running PrimerMapper.
